# The People’s Health Journal of Chicago

**Published:** 1890-12

**Authors:** 


The People's Health Journal of Chicago, October 15th, 1890. L. D. Rogers, A. M., M. D., and S. Ida Wright

Rogers, M. D., editors, 441 Dearborn Avenue, Chicago, Illinois. Price, $1.00 a year. Ten cents a copy.

This is a folio journal of eight pages, issued once a month, and devoted to hygiene, dietetics, and Homœopathy. It is an excellent publication, several of its contributors being contributors of THE HOMŒOPATHIC PHYSICIAN. Its articles are exceedingly instructive and will prove interesting not alone to the medical man but to his family as well. Its low price puts it within the reach of all. W. M. J.

